# Creatine Supplementation in Preclinical Models Receiving Immune Checkpoint Inhibitor Therapy: A Systematic Review

**DOI:** 10.7759/cureus.108384

**Published:** 2026-05-06

**Authors:** Jeffrey Shin, Raghavee Neupane, Gilberto Lopes, Atif Hussein, Tony Ricci, Marc M Kesselman

**Affiliations:** 1 College of Medicine, Nova Southeastern University Dr. Kiran C. Patel College of Osteopathic Medicine, Davie, USA; 2 Division of Medical Oncology, Department of Medicine, University of Miami Miller School of Medicine, Jackson Memorial Hospital, Miami, USA; 3 Department of Hematology/Oncology, Memorial Cancer Institute, Pembroke Pines, USA; 4 Department of Exercise and Sport Science, Nova Southeastern University, Davie, USA; 5 Department of Rheumatology, Nova Southeastern University Dr. Kiran C. Patel College of Osteopathic Medicine, Davie, USA

**Keywords:** cd8+ t cell activation, checkpoint inhibitor therapy, creatine supplement, pd-1 inhibitor, phosphocreatine

## Abstract

Creatine supplementation is widely used for its ergogenic benefits but has recently garnered interest for its immunomodulatory properties, particularly its capacity to enhance CD8+ T-cell bioenergetics through SLC6A8-mediated transport and phosphocreatine-dependent ATP (adenosine triphosphate) buffering. Emerging preclinical evidence suggests that creatine may potentiate antitumor immunity and augment the efficacy of immune checkpoint inhibitor (ICI) therapies. Conflicting reports of creatine-associated metastasis in select tumor models have raised uncertainty regarding its role in oncologic settings. This review synthesizes current preclinical evidence to assess whether creatine exerts beneficial, neutral, or detrimental effects across experimental oncologic models treated with immune checkpoint therapy. A total of 230 articles were screened, and 5 studies were included within this systematic review. Current preclinical evidence suggests that creatine supplementation may exert beneficial immunomodulatory and antitumor effects when combined with PD-1 blockade, largely by enhancing T-cell metabolic fitness and macrophage-driven inflammatory responses. However, the potential for metastasis in select tumor types poses a risk to the administration of a creatine supplement. This systematic review emphasizes the absence of human data, highlighting a critical knowledge gap. Rigorous mechanistic studies and early-phase clinical trials are essential to determine whether creatine can be safely and effectively integrated into ICI-based treatment strategies.

## Introduction and background

Creatine supplements are widely recognized for their role as effective ergogenic aids among athletes, bodybuilders, and fitness professionals. Their multifaceted benefits include enhanced anaerobic energy capacity, reduced protein breakdown, and increased muscle mass and physical performance [1]. Additionally, the clinical applications of creatine supplementation have been studied in contexts such as neurodegenerative diseases, diabetes, fibromyalgia, aging, and brain health [2].

Recent research has expanded interest in creatine’s immunological effects, particularly its ability to enhance CD8+ T-cell function [3]. Once creatine is consumed, it is transported into cells via the sodium-chloride-dependent transporter (SLC6A8) against a concentration gradient [4], where it is converted to phosphocreatine (PCr) and serves as a rapid phosphate donor for ATP [5]. The ATP (adenosine triphosphate) reservoir supports energy signaling in developing and mature T cells when they interact with the host major histocompatibility complex (MHC) [6]. Recent evidence has also identified creatine as a critical metabolic regulator of antitumor T-cell immunity, highlighting its potential benefits in cancer treatment [7]. Additionally, in nutrient-poor tumor microenvironments, T cells compete with tumors for essential nutrients, such as amino acids and glucose, making energy reservoirs even more crucial [8].

However, this theoretical benefit has not translated into measurable clinical improvements in patients with cancer. The American Society of Clinical Oncology states that creatine supplementation does not improve weight, appetite, quality of life, strength, or body composition based on randomized trials [9,10]. Recent experimental studies suggest that creatine may promote cancer metastasis in highly malignant cancers [11]. However, various preclinical studies suggest that creatine may enhance antitumor immunity by increasing ATP production in macrophages and CD8+ T cells and may increase the sensitivity of tumor cells to immunotherapy [7,11-13]. These findings are particularly relevant in the context of immune checkpoint inhibitors (ICIs), which have revolutionized cancer therapy by reactivating exhausted T cells. ICIs block inhibitory pathways such as CTLA-4 and PD-1 or PD-L1, which tumors exploit to suppress immune surveillance [14-17]. By administering monoclonal antibodies against CTLA-4 (ipilimumab) or PD-1 or PD-L1 (nivolumab, pembrolizumab, atezolizumab), checkpoint blockade enhances the proliferation of cytotoxic T lymphocytes (CTLs). This leads to the expansion and activation of tumor-infiltrating CD8+ T cells, thereby increasing interferon-gamma signaling and facilitating the lysis of cancer cells [18-20].

The existing body of scientific literature on this topic has been marked by considerable debate. Implementing this approach in clinical trials is a significant challenge. Nevertheless, as preclinical and clinical evidence continues to accumulate, creatine supplementation may demonstrate unrecognized therapeutic benefits, potentially redefining current paradigms in oncologic treatment strategies. This review aims to critically examine the impact of creatine supplements on immune checkpoint therapies in preclinical models.

## Review

Methods

A systematic literature review was performed using Ovid (MEDLINE), CINAHL, EMBASE, PubMed, and Web of Science, using the search terms “Immune checkpoint inhibitors” OR “PD-1” OR “PD-L1” OR “CTLA-4” OR “LAG-3” AND “Cancer” OR “Neoplasm” OR “Tumor” OR “Malignancy” AND “Immune therapy” OR “Immunotherapy” AND “Creatine” OR “Phosphocreatine” OR “Creatine supplementation.” To ensure article recency, only articles published in English between 2010 and 2025 were assessed. The articles were analyzed in a stepwise process, first evaluating the title and abstract for relevance and then assessing the full-text manuscript. Studies were excluded if they were non-English, published outside the specified date range, did not involve immune checkpoint inhibitor therapy or creatine supplementation, lacked relevance based on title or abstract screening, or did not provide sufficient full-text data; review articles, editorials, and conference abstracts without complete data were also excluded. The Nova Southeastern University (NSU) library database was utilized to access databases and full-text articles.

Eligible studies included cell line or animal model studies across any cancer type, provided ICI treatment (PD-1, PD-L1, CTLA-4, or LAG-3 inhibitors) was part of the therapeutic regimen. The intervention of interest was creatine supplementation (oral, intravenous, or otherwise administered) during or after ICI therapy, with comparisons made to placebo, standard care, or control groups receiving ICI therapy without creatine. Primary outcomes included clinical measures such as overall survival, progression-free survival, cancer progression, immune-related effects, and treatment-related adverse events. Preclinical animal studies were included and analyzed for tumor response (e.g., tumor volume and growth kinetics), survival outcomes, immune cell infiltration and activation (including CD8+ T-cell and macrophage activity), relevant molecular and metabolic pathways, and any reported adverse or off-target effects of creatine supplementation in the context of immune checkpoint inhibitor therapy. Eligible study designs included relevant preclinical or in vivo research. Studies were excluded if they did not involve immune checkpoint inhibitor therapy, did not assess creatine supplementation as an intervention, or failed to report relevant outcomes. In addition, review articles, editorials, conference abstracts without full data, and non-English-language publications were excluded.

**Figure 1:**
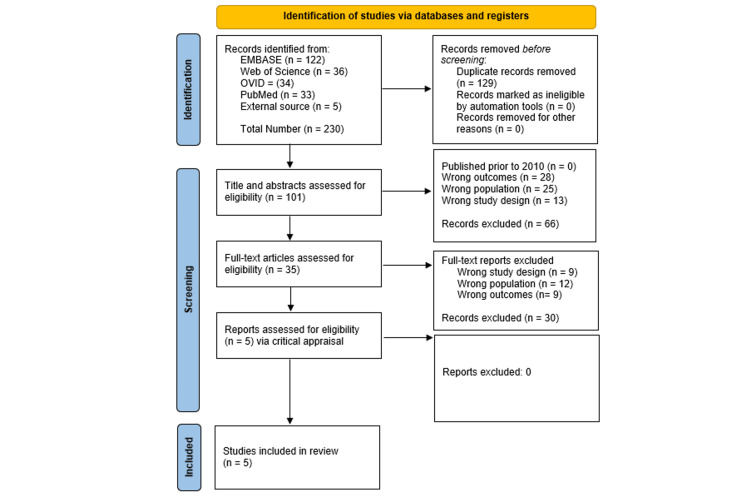
PRISMA flow diagram indicating data selection PRISMA: Preferred Reporting Items for Systematic Reviews and Meta-Analyses

Two reviewers completed a blinded review of the articles to determine inclusion or exclusion based on the established criteria, and a third reviewer was used to resolve any disagreements. Quality assessment of the included studies was conducted using the Joanna Briggs Institute Critical Appraisal Tools, with studies categorized as low (>70%), moderate (50-70%), or high (<50%) risk of bias; discrepancies were resolved through discussion or consultation [21]. All papers met the inclusion criteria, and the Preferred Reporting Items for Systematic Reviews and Meta-Analyses (PRISMA) guidelines were followed and used to develop a flow diagram of the selection criteria for reproducibility (Figure 1) [22].

Results

The initial search using the strategy across four databases yielded 230 articles. Following deduplication, 129 duplicates were excluded, leaving 141 unique articles that were screened based on the title, abstract, and study design. A total of five full-text articles were included in the final extraction results [7,12,23-25]. Table 1 summarizes the findings of the included studies, including test subjects, cancer type, the effect of creatine on immune cell modulation, effects on ICI, effects on tumor, and the molecular signaling pathway (Table 1). 

**Table 1:** Findings of the studies analyzed in the review CRC: colorectal cancer, PD-1: programmed cell death protein 1, IFN: interferon, SLC6A8: a gene encoding the creatine transporter protein, ERK2: extracellular signal-regulated kinase 2, MEK-1: mitogen-activated protein kinase kinase 1, CrT: creatine transporter, PCr: phosphocreatine, ATP: adenosine triphosphate, NSG: NOD scid gamma, MPS1: mucopolysaccharidosis type 1, Smad: suppressor of mothers against decapentaplegic, GATM: glycine amidinotransferase, TGF: transforming growth factor, TCR: T-cell receptor, CK: creatine kinase, ICI: immune checkpoint inhibitor, SHP2: Src homology region 2-containing protein tyrosine phosphatase 2.

Study	Year of publication	Study design	Cell lines/animal model	Type of cancer	Immune cell modulation	Source of creatine	Effect on ICI	Effect on tumor	Molecular signaling pathway	Clinical correlation
Di Base et al. [7]	2019	Pre-clinical experimental study that uses in-vivo mouse models	C57Bl.6 mice	Melanoma and colon adenocarcinoma	Increased expression of CrT. Creatine provides a readily available high-energy reservoir for ATP	Creatine monohydrate intraperitoneal injection and dietary supplementation	The combination of creatine supplementation and anti-PD-1 treatment generated significant tumor suppression	Combination therapy completely eradicated their tumor. Monotherapy with creatine saw T-cell dependent therapeutic effect	TCR activation increases CrT mRNA and protein in CD8 T cells. The CK system buffers intracellular ATP to power T cell activities	Creatine could be used as an adjunct therapy in clinical oncology settings to enhance ICI
Peng et al. [12]	2023	Pre-clinical mechanistic study, combining an in-vivo murine tumor model with in-vitro cellular assay	8- to 12-week old C57BL/6 mice	Murine melanoma using B16-F10 cell line	Increased intratumoral macrophage frequency and number, Pro-inflammatory cytokine gene expression, and Antigen-presenting capacity of macrophages. Antigen-specific CD8+ T cells,	Creatine was synthesized from glycine and L-arginine with daily intraperitoneal injection	Enhanced CrT blockade	Significantly suppressed B16-F10 tumor growth and volume	Increased intracellular ATP in macrophages by elevating PCr and creatine kinase, mediated by the cytosolic PCr system.	Enhance anti-tumor immunity by boosting macrophage ATP and function.
Zhou et al. [23]	2025	Multistep in-vitro mechanistic assay	Human CRC cell lines DLD-1 and SW480; human embryonic kidney. DLD-1 xenografts in BALB/c nude mice. MC38 CRC model using PD-1 combination.	CRC	Increased tumor-infiltrating CD8+ T cells in MC38 tumors, elevated IFN-gamma and granzyme B and boosting cytotoxic T cell function	Predominantly extracellular uptake via SLC6A8	Increase in infiltrating CD8+ T cells after creatine treatment, resulting in higher levels of IFN-γ and granzyme B	Inhibited tumor growth in DLD-1 xenograft and extended survival in mice model	SLC6A8 transports creatine into CRC, where it acts as a signaling metabolite. Directly binds to ERK2, impairs MEK1-mediated ERK2 activation.	SLC6A8 protein levels are reduced in tumors. ERK2-FSP1 and augments CD8+ T cell
Zhang et al. [24]	2021	Pre-clinical, mechanistic experimental study	Orthotopic mouse models using both immunodeficient NSG mice and immunocompetent BALB/c mice, Human CRC, mouse CRC, and mouse breast cancer cell lines	Colorectal cancer with a focus on liver metastasis; murine breast cancer	Metastasis-promoting effect of creatine was observed in NSG mice through MPS1-mediated Smad2/3 activation	Dietary supplementation (5% w/w creatine diet), upregulated de novo synthesis via GATM in metastatic lesions	Not reported	Creatine did not affect or reduce primary tumor growth in orthotopic CRC and breast models, increased liver metastasis of CRC, and lung metastasis of breast cancer	Creatine activates Smad2/3 phosphorylation via mitotic kinase MPS1, independent of TGF-B receptor signaling.	GATM is significantly upregulated in CRC liver metastases. Targeting GATM reduced metastasis and with combine with 5-fluorouracil
Homet Moreno et al. [25]	2016	Pre-clinical, mechanistic animal study that uses syngeneic murine tumor models	Immunocompetent mice	Melanoma and colon adenocarcinoma	Activity of T cells was crucial. The antitumor activity of PD-1 blockade required CD4+ and CD8+ cells. Depletion of CD4+ T cells completely reversed the antitumor effect in both models	Not reported	PD-1 blockade therapy required CD4 and CD8 T cell function and also require the costimulation of CD28 and CD80/86	Effects on tumor were diminished when CD4 T cells, or both CD4 and CD8 cells, were depleted	PD-1 forms a negative costimulatory microcluster that recruits the phosphatase SHP2, directly dampening TCR signaling. PD-L1 engagement can drive ligand-induced TCR down modulation on CD8 T cells	Patients whose tumors already exhibit an "inflamed" microenvironment are more likely to benefit from PD-1 therapy

Among the included studies, creatine supplementation was evaluated for its effects on ICI efficacy and tumor progression. Notably, Di Base et al. used CD4+ and CD8+ knockout mice to demonstrate that T cells are essential for both creatine supplementation and ICI therapy [7]. All studies concluded that creatine supplementation has a synergistic effect with PD-1 blockade therapy, with the exception of Zhang et al., who advised against creatine use due to observed increases in liver and lung cancer metastases [24]. Within this study, mice were given dietary and water supplementation of creatine at 5% w/w creatine monohydrate and 42.5 mg/mL of creatine, respectively. It is important to note that no clinical trials have been conducted evaluating creatine supplementation in cancer patients due to potential oncologic risks. Furthermore, the study reported that creatine did not affect or reduce primary tumor growth.

All four studies, except Zhang et al., provided detailed analyses of creatine’s effects on ICI therapy and tumor burden [7,12,23-25]. Specifically, Di Base et al. highlighted that creatine supplementation significantly suppressed B16-OVA tumor growth (p<0.0001) and reduced PD-1hiCD62Llo cells (p<0.01) [7]. Creatine supplementation synergized with anti-PD-1 to achieve superior tumor control, with most mice eradicating tumors and developing a memory response upon remission. The mean tumor size decreased from 45 mm² to 8 mm² with creatine supplementation by day 17 (p<0.0001) [7]. Similarly, Peng et al. reported that creatine reduced B16-F10 melanoma tumor volume from 588.5 ± 52.8 mm³ to 377.6 ± 35.8 mm³ by day 14, with statistical significance defined at p<0.05, p<0.01, and p<0.001 [12]. Creatine also increased intratumoral macrophage frequency and promoted an inflammatory M1 phenotype, as confirmed by t-test and ANOVA analyses (p<0.05 to p<0.001) [12].

**Figure 2:**
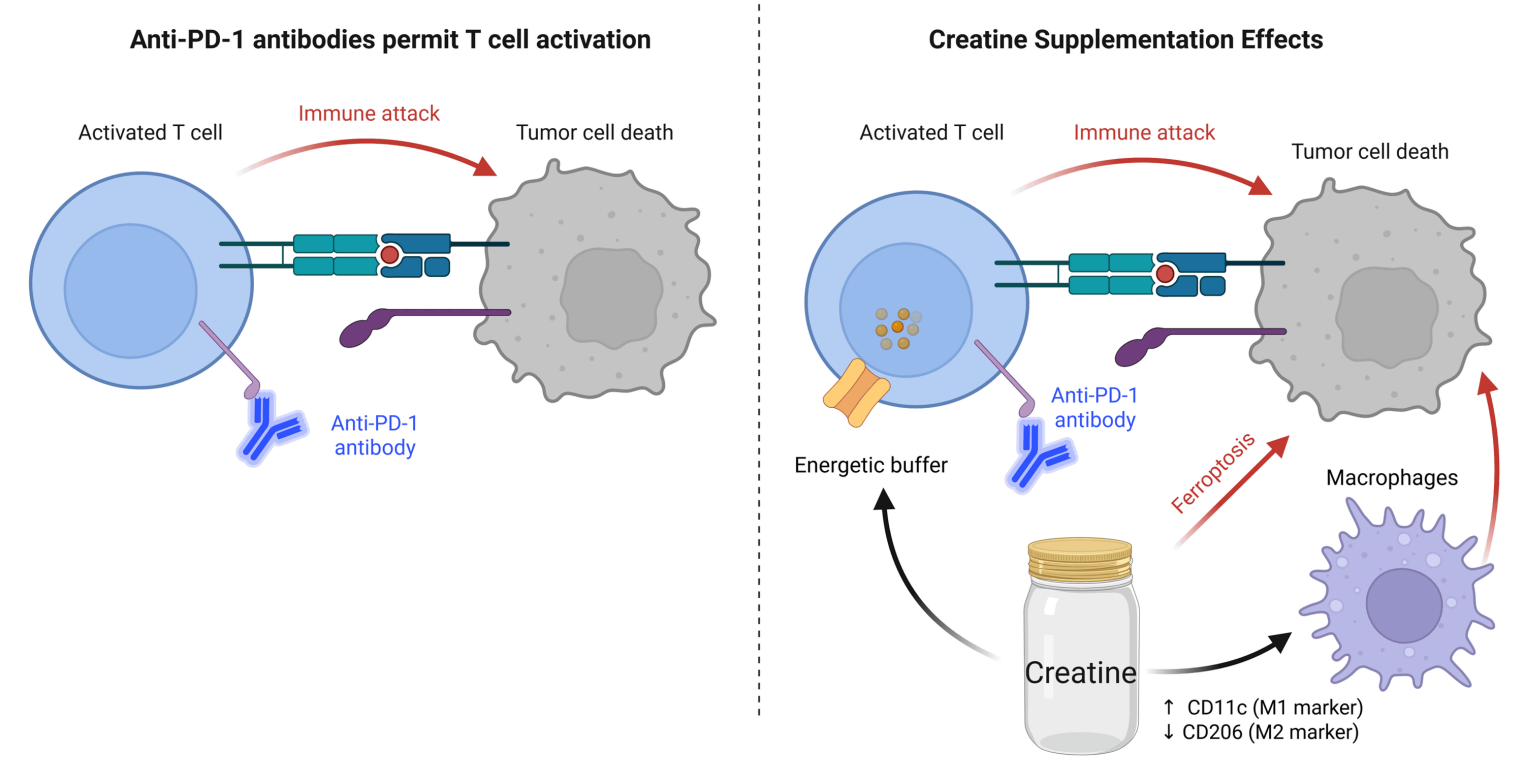
Creatine supplement augments antitumor immunity Created by the authors using BioRender. Creatine augments antitumor immunity through three coordinated pathways: (1) acting as an energetic buffer in cytotoxic CD8⁺ T cells to sustain effector function and tumor cell killing; (2) sensitizing tumor cells to ferroptosis via suppression of the SLC6A8–ERK2–FSP1 axis, reducing ferroptosis defense mechanisms; and (3) promoting macrophage polarization toward a pro-inflammatory M1-like phenotype, marked by increased CD11c and decreased CD206 expression. Together, these mechanisms support enhanced immune-mediated tumor regression during ICI therapy.

In another study, Zhou et al. found that creatine supplementation reduced mean tumor weight from 1.4 g in controls to 0.3 g in the treatment group [23]. Additionally, the study found that CD8+ T cells increased from 29.4% to 46.9% (p=0.00012). Combining creatine with anti-PD-1 therapy markedly suppressed MC38 tumor growth, reduced tumor weight, and prolonged survival compared with monotherapy [23]. Moreno et al. did not primarily investigate creatine supplementation; their findings underscored that the in vivo efficacy of anti-PD-1 therapy depends on both CD4+ and CD8+ T cells, and that creatine supplementation may help maintain T-cell bioenergetic function [25] (Figure 2).

In contrast, Zhang et al. used orthotopic mouse models and showed that creatine supplementation significantly enhanced liver metastasis in HCT116 (p=0.0317) and CRC57 (p=0.0079) mouse models, with reduced survival times in both HCT116 (p=0.025) and CRC57 (p=0.0019) [24]. However, these experiments employed severely immunocompromised mice. As highlighted by Moreno et al., the immune microenvironment is a critical determinant of both PD-1 inhibitory therapy and the potential immunomodulatory benefits of creatine supplementation [25]. A summary of the quality assessment using the Joanna Briggs Institute Critical Appraisal Tool is presented in Table 2 (Table 2) [21]. 

**Table 2:** Quality assessment using the Joanna Briggs Institute critical appraisal tool Risk of bias for each included study was assessed using the Joanna Briggs Institute Critical Appraisal Tool. Risk of Bias was assessed under three categories: selection bias, performance/detection bias, reporting and other bias. An overall quality rating was made based on the assessment.

Study	Risk of selection bias	Performance/detection bias	Reporting and other bias	Overall quality rating
Di Base et al. [7]	Moderate	Low-moderate	Moderate	Moderate
Peng et al. [12]	High	Low	Low-moderate	Moderate
Zhou et al. [23]	Low-moderate	Moderate	Low	Low-moderate
Zhang et al. [24]	Low-moderate	Low-moderate	Moderate	Moderate
Moreno et al. [25]	Moderate	Moderate	Low-moderate	Moderate

Discussion

Creatine supplementation has seen a recent surge in popularity among athletes and recreational weightlifters as a dietary supplement to increase intramuscular creatine and enhance energy availability [26-28]. Athletes have used a loading phase of approximately 0.3 grams per kilogram, followed by daily doses of 5 grams for maintenance and 10 grams for larger athletes. Noting that 95% of creatine storage occurs in skeletal muscle may provide insight for physicians considering supplementation as an adjunct therapy, independent of delivery [2]. Similarly, scientific evidence has shown increased interest in creatine supplements and their benefits for older adults, individuals with dietary restrictions, women, and patients with neurodegenerative or metabolic diseases [2,28,29]. While still speculative, it has been proposed that dosing for improved cognition and as an aid in neurodegenerative disease is at a higher threshold than needed for performance; it appears that dosages of 20 grams and above have shown the most consistent results in increasing creatine levels in healthy populations. Such dosing may be a caveat, as noted by physicians for patients susceptible to certain cancers [30]. The mechanism by which creatine supplements may positively affect ICI therapy is similar to that of a central energy reserve, enabling immune responses to function effectively. Cancer cells utilize the body’s energy sources to metastasize, leaving the body and the immune system with limited resources to effectively fight the disease.

As mentioned previously, ICI therapy treats cancer by blocking inhibitory signals delivered by CTLA-4 or PD-1 or PD-L1, thereby enhancing T-cell proliferation, cytokine production, and cytotoxic activity within the tumor microenvironment [18,31,32]. Creatine supplementation may enhance the efficacy of ICI therapy for cancer by supporting T-cell and macrophage bioenergetics, thereby amplifying antitumor responses. Creatine also promotes ATP production in macrophages, thereby enhancing antigen presentation and increasing tumor antigen-specific CD8+ T-cell responses [7,12,13]. These findings suggest a mechanistic rationale for combining creatine supplementation with ICI therapy to boost immune-mediated tumor control. Creatine has been shown to affect glycemic control; whether such metabolic effects have any relevance to cancer outcomes is unknown [33-35]. Another potential benefit of using creatine supplements as an adjunct to cancer therapy is a reduction in sarcopenia, the progressive loss of skeletal muscle mass and function [36,37]. Sarcopenia may impair the efficacy of ICIs by promoting chronic inflammation, altering immune cell function, and reducing the host’s ability to mount an effective antitumor immune response [38,39]. Progressive loss of muscle mass during ICI therapy further worsens prognosis and may reflect aggressive tumor biology, thus emphasizing the potential benefit of creatine supplementation in cancer patients [36,37,40].

The kidney plays a crucial role in endogenous creatine synthesis via the enzyme arginine:glycine amidinotransferase (AGAT) [41]. Consequently, patients with chronic kidney disease (CKD) experience a progressive decline in endogenous creatine production, predisposing them to systemic creatine deficiency [41]. Despite this, creatine supplementation in individuals with renal impairment warrants careful consideration, as exogenous creatine imposes an additional metabolic burden on already compromised renal function [42,43]. In cancer patients with underlying renal disease, creatine supplementation may therefore pose risks related to accelerated kidney dysfunction, as well as impaired renal clearance of excess creatine and its metabolic byproduct, creatinine [42,44].

The most significant limitation of this review is that minimal research has been conducted on this topic, especially in clinical settings. This may reflect concerns regarding the potential for creatine supplements to exacerbate metastasis in certain cancers, creating an ethical dilemma while potentially placing patients at risk of further complications. The increased risk of cancer metastasis has been observed in certain contexts, but the evidence is mixed and largely preclinical. Recent studies have shown that creatine can promote metastasis in colorectal, breast, and pancreatic cancers by activating the MPS1-Smad2/3 signaling axis, leading to upregulation of pro-metastatic factors such as Snail and Slug [24,45]. Therefore, caution is warranted when considering creatine supplementation in patients with active or high-risk cancers. However, other studies included in this review indicate that creatine supplementation can have antitumor effects, such as inducing ferroptosis in colorectal cancer cells, enhancing antitumor immunity, and increasing the efficacy of ICI therapy in mouse models [7,12,13,23,46]. Clinical and animal studies have not shown increased tumor growth or aggressiveness with creatine supplementation, and some have reported neutral or even beneficial effects [46,47].

## Conclusions

While preclinical studies suggest that creatine supplementation may augment the efficacy of immune checkpoint inhibitor therapy, the absence of robust clinical evidence limits the ability to draw definitive conclusions regarding its therapeutic role. The existing data are predominantly preclinical and highlight important context-dependent risks, including the potential to promote metastasis in certain tumor types. These uncertainties underscore the need for rigorously designed clinical trials and mechanistic studies to clarify safety, define patient selection criteria, and determine optimal dosing strategies. As the field advances, creatine supplementation may emerge as a safe, accessible, and biologically rational adjunct to ICI therapy, but its integration into clinical practice must be guided by evidence and careful oncologic evaluation.
